# Diversity patterns of ground beetles and understory vegetation in mature, secondary, and plantation forest regions of temperate northern China

**DOI:** 10.1002/ece3.1367

**Published:** 2015-01-07

**Authors:** Yi Zou, Weiguo Sang, Shunzhong Wang, Eleanor Warren-Thomas, Yunhui Liu, Zhenrong Yu, Changliu Wang, Jan Christoph Axmacher

**Affiliations:** 1 UCL Department of Geography, University College LondonLondon, WC1E 6BT, UK; 2 The State Key Laboratory of Vegetation and Environmental Change, Institute of Botany, Chinese Academy of SciencesBeijing, 100093, China; 3 College of Life and Environmental Science, Minzu University of ChinaBeijing, 100081, China; 4 College of Resources and Environmental Sciences, China Agricultural UniversityBeijing, 100193, China

**Keywords:** *α*-Diversity, biodiversity conservation, carabids, herbaceous plants, mature forest, turnover

## Abstract

Plantation and secondary forests form increasingly important components of the global forest cover, but our current knowledge about their potential contribution to biodiversity conservation is limited. We surveyed understory plant and carabid species assemblages at three distinct regions in temperate northeastern China, dominated by mature forest (Changbaishan Nature Reserve, sampled in 2011 and 2012), secondary forest (Dongling Mountain, sampled in 2011 and 2012), and forest plantation habitats (Bashang Plateau, sampled in 2006 and 2007), respectively. The *α*-diversity of both taxonomic groups was highest in plantation forests of the Bashang Plateau. Beetle *α*-diversity was lowest, but plant and beetle species turnover peaked in the secondary forests of Dongling Mountain, while habitats in the Changbaishan Nature Reserve showed the lowest turnover rates for both taxa. Changbaishan Nature Reserve harbored the highest proportion of forest specialists. Our results suggest that in temperate regions of northern China, the protected larch plantation forest established over extensive areas might play a considerable role in maintaining a high biodiversity in relation to understory herbaceous plant species and carabid assemblages, which can be seen as indicators of forest disturbance. The high proportion of phytophagous carabids and the rarity of forest specialists reflect the relatively homogenous, immature status of the forest ecosystems on the Bashang Plateau. China's last remaining large old-growth forests like the ones on Changbaishan represent stable, mature ecosystems which require particular conservation attention.

## Introduction

The loss of biodiversity due to deforestation has been of increasing global concern. Only 36% of the world's forest cover of 4 billion ha consists of “primary” or mature forests, whereas 53% of the forest ecosystems are modified natural forests, 7% represent seminatural forests and 4% are forest plantations (FAO 2006). Secondary and plantation forests are therefore becoming an increasingly important component of global forest cover (Bass 2004; FAO 2006; Liu et al. 2008). Although it is believed that mature forests are irreplaceable for sustaining biodiversity (Gibson et al. 2011; Ruiz-Benito et al. 2012), secondary and plantation forests are known to play an important role in maintaining forest biodiversity (Barbaro et al. 2005). The overall contribution secondary and plantation forests make toward biodiversity conservation nonetheless has been vigorously debated (Lawton et al. 1998; Brockerhoff et al. 2001, 2008; Hartley 2002; Sayer et al. 2004; Gibson et al. 2011).

China currently harbors the world's largest plantation forest cover, totaling about 62 million ha. This represents about 31.8% of China's total forest area (Chinese State Forestry Bureau 2011), with large-scale planned afforestation and reforestation programs expected to further extend this area in the near future (Wang et al. 2007). Not least due to the marked regeneration of native forest vegetation in the undergrowth of many forest plantations, any logging activities beyond normal stand management have been strictly banned in these forests (Wang et al. 2007), generating a number of large-scale, well-protected plantation and secondary forest mosaics (Li 2004). Their protection is further underpinned by various government programs aimed at conserving reforestation and afforestation forest resources. The main functions of these large-scale protected secondary forests and forest plantations are seen in the prevention of soil erosion and associated sand storms as well as in carbon sequestration. Their establishment, however, is lacking clear conservation and habitat restoration objectives (Sayer et al. 2004). In some cases, forest plantations have even resulted in substantial ecosystem and biodiversity degradation (Cao et al. 2010b; Xu 2011), while in most cases, their effects on local species richness and composition are unknown.

Previous studies comparing the biodiversity of mature, secondary, and plantation forests are mostly limited to small sites in relatively limited overall study areas (Elek et al. 2001; Carnevale and Montagnini 2002; Maeto et al. 2002; Barlow et al. 2007a,b; Makino et al. 2007). One potential problem of such comparisons is the relative vicinity of the different forest types (Carnus et al. 2006; Chazdon et al. 2009), as transient species particularly in mobile insects may be infrequently recorded among each forest type (Barlow et al. 2007b). It is also often difficult to avoid the impact of edge effects in diversity studies based in finely grained forest mosaics (Lopez-Barrera et al. 2005; Pardini et al. 2009). Accordingly, to enhance the general understanding of the role plantation and secondary forests play in biodiversity conservation, an additional comparison between mature, plantation, and secondary forests distributed across wider geographical areas will have great significance. Here, we selected three distinct study regions in the temperate region of north China, with forests of these regions being clearly dominated by mature, secondary, and plantation forest ecosystems, respectively. As it is technically impossible to assess the complete diversity status of all species in forest ecosystems, a more feasible alternative is to focus research on some of the most species-rich taxonomic groups. Here, we selected a highly diverse ground-dwelling arthropod group, ground beetles (Coleoptera: Carabidae). Ground beetles are very sensitive to environmental change and have been successfully used as bio-indicators in investigations into the regional biodiversity status in relation to forest management in Europe (Humphrey et al. 1999; Magura et al. 2000, 2001; Koivula 2002; Finch 2005; Lövei et al. 2006; Karen et al. 2008; Lange et al. 2014; Negro et al. 2014), Africa (Rainio and Niemelä 2006), Central and North America (Klimaszewski et al. 2005; Ulyshen et al. 2006), and south China (Meng et al. 2012). In addition to ground beetles, the composition and diversity of plant species were also recorded in all three study regions. Given that the main woody plant species have been planted in plantation forests and in some parts of secondary forests in our study regions, we did not consider them to be suitable indicators of phyto-diversity and focused our vegetation surveys on understory herbaceous plant assemblages, as this group contains the main diversity of the vegetation.

The overall objectives of this study were to compare the *α*-diversity of herbaceous plants and carabids in the three regions dominated by mature, secondary, and plantation forests, respectively, and to analyze the structure of ground beetle and plant assemblages in the study regions. We hypothesize that the mature forest harbors the highest diversity for both carabids and plants, followed by the secondary forest, with the plantation forest harboring the lowest diversity levels. This is based on our expectations that particularly forest specialist species will only have survived in China's remnant mature forest ecosystems and that the relatively homogeneous, monodominant canopy cover encountered in plantation forests will only support a limited diversity in both, understory plant and ground beetle species. Furthermore, we hypothesize that the species composition varies according to the study regions, but expect a notable overlap particularly in widespread, generalist species between the different regions, with both mature forest and forest plantation hypothesized to share more species with the secondary forest region than with each other.

## Materials and Methods

### Study regions

**Figure 1:**
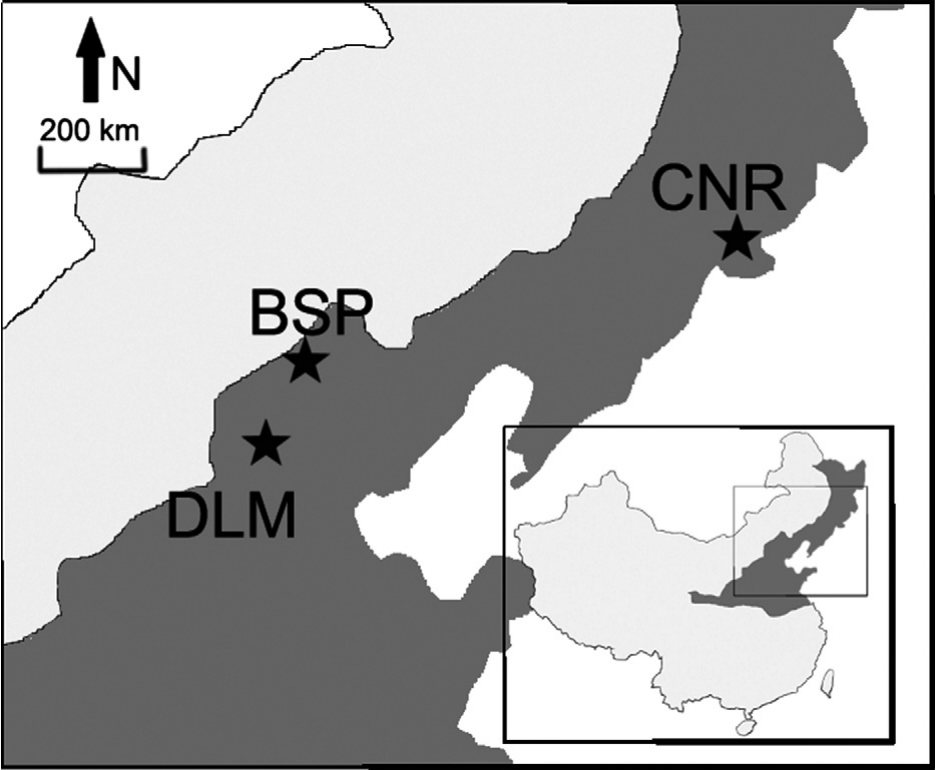
Map of the study region, with the gray areas showing the extend of the temperate forest region (BSP: Bashang Plateau; CNR: Changbaishan Nature Reserve; DLM: Dongling Mountain).

Our study areas are located at three distinct geographical regions that harbor different forest types. The first study region was located in the Changbaishan Nature Reserve (CNR) (41°41′–42°51′N, 127°43′–128°16′E) in Jilin Province (Fig. 1). The reserve has an area of about 20,000 ha, making it the largest pristine temperate forest region in northern China. Four different forest zones can be clearly distinguished with increasing elevation (Chen et al. 1964; Xu et al. 2004; Sang and Bai 2009): a mixed coniferous and broad-leaved forest zone at 700–1100 m, a mixed coniferous forest zone at 1100–1500 m, a subalpine mixed coniferous forest zone at 1500–1800 m, and a birch forest zone at 1800–2100 m. Based on similarities in climatic conditions across the study regions investigated here, we selected 11 study plots in the continuous mixed coniferous and broad-leaved forest area for this study. These forests experience an average annual temperature of 3.4°C, and the annual precipitation amounts to 654 mm (Chen et al. 1964; Sang and Bai 2009).

The second study region was located at the Dongling Mountain (DLM) (40°00′N, 115°26′E), which is located about 110 km southwest of the Beijing city center near the boundary between Beijing and Hebei Province (Fig. 1). The altitude of the mountain ranges from 800 m to 2300 m. The region was originally dominated by oak (*Quercus liaotungensis* Mayr) forests, but was extensively deforested and large areas were subsequently recolonized by secondary forests, while other areas were partly reforested with pine (*Pinus tabulaeformis* Carrière) and larch (*Larix principis-rupprechtii* Mayr) plantations in the 1960s (Yu et al. 2010). This resulted in a finely grained mosaic of different interlinking forest types covering the area. Our 12 selected plots at DLM were located at Beijing Xiaolongmen Forest Park and represented forest patches of secondary oak, birch (*Betula platyphylla* Suk. and *B. dahurica* Pall.), and mixed forests, with four plots selected in each of these forest types. This region experiences a warm temperate and continental monsoon climate, with an annual mean temperature at 1100 m of 4.8°C and an average annual precipitation of 612 mm (Sang 2004).

The final research region was located on the Bashang Plateau (BSP) (Fig. 1) in the mountain ranges of the Hebei Province between Beijing and the Inner Mongolian Plateau, which represents the transition zone between the subhumid monsoon climate and the semi-arid steppe climate (Zhao et al. 2005). The vegetation therefore falls within the boundary between the warm temperate deciduous broad-leaved forest zone and the temperate grassland zone (Zhang 2007). In the past, the region has experienced severe land degradation due to overgrazing and forest transformations into cropland (Zhao et al. 2005). Since the late 20th century, a wide range of forest plantations has been established in this region under policies such as the “Grain for Green” Project and the “Sand Control Programme” (Cao 2011), with larch (*Larix principis-rupprechtii* Mayr) as the main plantation tree species. In this region, we selected separate forest plantations of varying sizes in two main areas, Baiqi Village (41°3′N, 116°11′E) at an elevation of about 1380 m asl and Shizigou Village (41°13′N, 115°23′E) at about 1650 m asl, where we selected 4 sampling plots, respectively. Apart from one plot in Shizigou which was a poplar (*Populus tomentosa* Carr) plantation, all plots were larch monocultures. The annual mean temperature at Baiqi ranges between 4°C and 6°C, with an average annual precipitation of 515 mm, whereas the annual mean temperature at Shizigou ranges between 2°C and 4°C, with annual precipitation below 500 mm.

These three study sites were therefore located in regions at a minimum distance exceeding 130 km. Plots were subsequently selected at different altitudes in each region to compensate for the different general temperature regimes associated with the change in latitude. Nonetheless, some differences in climatological conditions prevailed, which might also alter the biogeographical composition of the vegetation and the carabid assemblages. BSP sites in particular experience a wider amplitude of annual mean temperatures and are more severely affected by drought than sites at the other regions. Despite these limitations, the approach we used here based on three distinct regions addresses a lot of the limitations of studies using sites located in close proximity which we outlined above, and as all three regions are representative of the temperate forest zone of northern China (Zhang 2007), our study provides highly relevant and important information on the biodiversity status of these distinctive forest types.

### Insect sampling and vegetation survey

Not least due to differences in topography and accessibility, sampling approaches differed slightly between the sampling regions, as sampling was conducted during three different campaigns.

Carabids were all sampled using pitfall traps at individual plots with a minimum distance of 60 m between neighboring plots at all sites. In CNR, each study plot had a size of 20 × 20 m^2^ and was divided equally into four subplots, where four pitfall traps were, respectively, placed in the center of each subplot. Study plots also measured 20 × 20 m^2^ in DLM, while two pitfall traps at a distance of 2 m were randomly located inside the plot. Plots again measured 20 × 20 m^2^ in BSP. Here, an array of eight traps was positioned inside each plot. All pitfall traps were covered by an aluminum roof to protect samples from rainfall and leave litter contamination.

Due to the large geographical extend of the study regions and associated logistics, it was impossible to collect samples and set traps at all plots on the same day. In CNR, carabids were sampled continuously between June and August in 2011 and 2012, covering a sampling period between 11 and 14 weeks in the 2 years. In DLM, sampling was conducted between June and August in 2011, and between June and September in 2012 over continuous sampling periods totaling about 20 weeks. Carabids were sampled between May and October in 2006 and 2007 at the BSP. Nonetheless, sampling was limited to 6 days each month, resulting in a total sampling period of about 10 weeks. Overall, sampling period-related variations cannot be fully negated when comparing the carabid assemblages across the different regions (Harvey et al. 2008), although the sampling was conducted over 2 years at each region to account for interannual variability. The slightly longer sampling period used at BSP may also have increased the chance of catching more species than at the other two regions (Yu et al. 2006). This was partly related to the local climatic conditions and fire risk at CNR and DLM, which prevented sampling in late autumn. While we acknowledge that these different sampling regimes used at the three study regions might result in the under-representation particularly of some autumnal species, we are confident that our samples are widely representative of the carabid assemblages in China's temperate forests and cover the peak carabid activity periods in these forests at all three regions (Yu et al. 2002).

The presence of herbaceous species was recorded on four plots of 1 m^2^ randomly located within the subplots at CNR and DLM, and on five 2 × 2 m^2^ subplots located at the four corners of each plot and in the plot center at BSP. More detailed descriptions of the sampling designs at CNR can be found in Zou et al. (2014), at DLM in Warren-Thomas et al. (2014) and at BSP in Axmacher et al. (2011).

### Data analysis

To minimize between-sample variations in the same study region, data were pooled for *α*-diversity analysis. It needs to be noted that although BSP contains two distinct study areas, species turnover between these two areas was low for both carabids and herbaceous plants (see Results section), indicating relatively homogeneous species assemblages to occur across the two areas, and data within the BSP plots were therefore deemed suitable to be combined.

As the overall sampling efforts varied between different study regions, we used Hurlbert rarefaction to standardize and allow for direct comparison of the species richness of carabids. Hurlbert rarefaction is a proved approach allowing robust standardized comparisons of samples varying in sample sizes and underlying sampling effort (Hurlbert 2004; Olszewski 2004; Colwell et al. 2012; Fiedler and Truxa 2012). We selected individual-based rarefaction for carabids rather than sample-based (trap-based) rarefaction because different numbers of samples were used per plot in the different study areas as outlined above, and we believe that the number of specimens caught by each trap increases in a nonlinear fashion with an increasing number of traps. It is probable that the larger vegetation survey areas per plot sampled at BSP contain more species (Magurran 1988). Therefore, area-based rarefaction for incidence data was used for herbaceous plants to take full account of the difference in overall survey area sampled in the different study regions. Additionally, species rank–abundance plots were calculated to compare species dominance patterns of carabids between study regions.

In addition, we established the composition of carabid feeding guilds (carnivores, omnivores, or phytophagous) following the classifications of Harvey et al. (2008), Yu et al. (2010), Oelbermann and Scheu (2010), ElSayed and Nakamura (2010), Zhu et al. (1999), Hering and Plachter (1997) and based on further consultations with experts from the Institute of Zoology, Chinese Academy of Sciences. In cases where detailed information was missing, species from the same genus were considered as belonging to the same feeding guilds.

Beta-diversity can be defined in a number of ways (Tuomisto 2010). In the context of this study, we use the term to describe the degree of species turnover between different sampling plots (Whittaker 1960). To analyze this turnover, we calculated dissimilarity matrices reflecting changes in the species composition between plots for each taxon. For carabids, the dissimilarity matrices were calculated based on chord-normalized expected species shared (CNESS) for a minimum sample size (*m *=* *1), strongly weighing changes in the dominant species. The matrices for herbaceous plants were calculated based on the Jaccard index using incidence (presence–absence) data. Nonmetric two-dimensional scaling (NMTDS) was used to subsequently visualize the dissimilarity patterns.

CNESS results were calculated using the software COMPAH (Gallagher 1998). The remaining calculations and statistics were computed in R (R Development Core Team 2011), using the package “vegan” (Oksanen et al. 2012) to calculate the “Jaccard” index and to calculate the ordination plots.

## Results

### *α*-diversity

**Figure 2:**
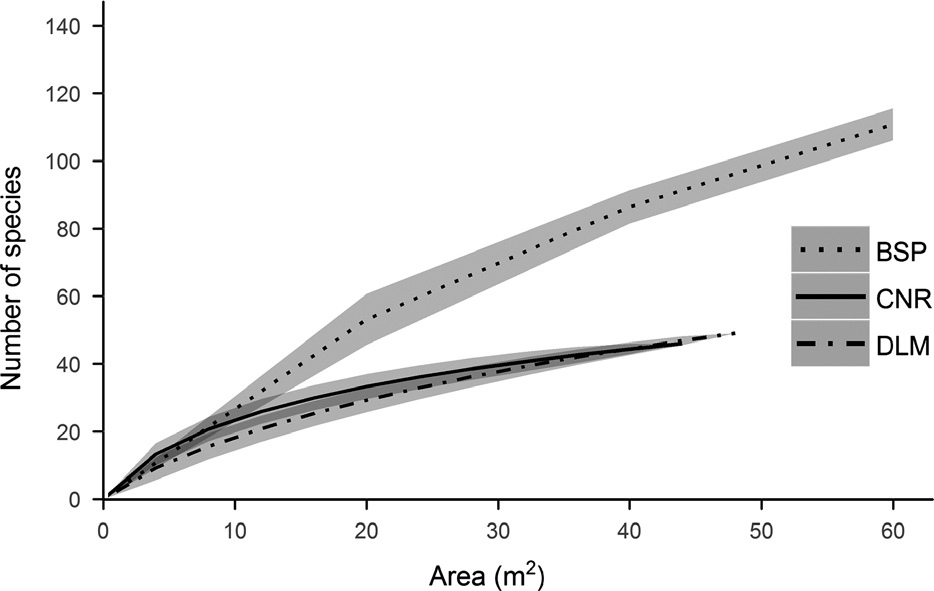
Species–area rarefaction curves of herbaceous plant species in the different study areas (shaded areas represent 95% confidence intervals; BSP: Bashang Plateau; CNR: Changbaishan Nature Reserve; DLM: Dongling Mountain).

In total, the three study sites contained 279 herbaceous plant species. Of these, 46 species were recorded in CNR and 49 were observed in DLM, whereas 184 species were recorded in BSP. Incidence-based species–area rarefaction showed that BSP was substantially more diverse in the number of herbaceous plant species than the other two regions. Rarefaction curves furthermore showed a similar number of herbaceous plant species at both CNR and DLM (Fig. 2).

**Figure 3:**
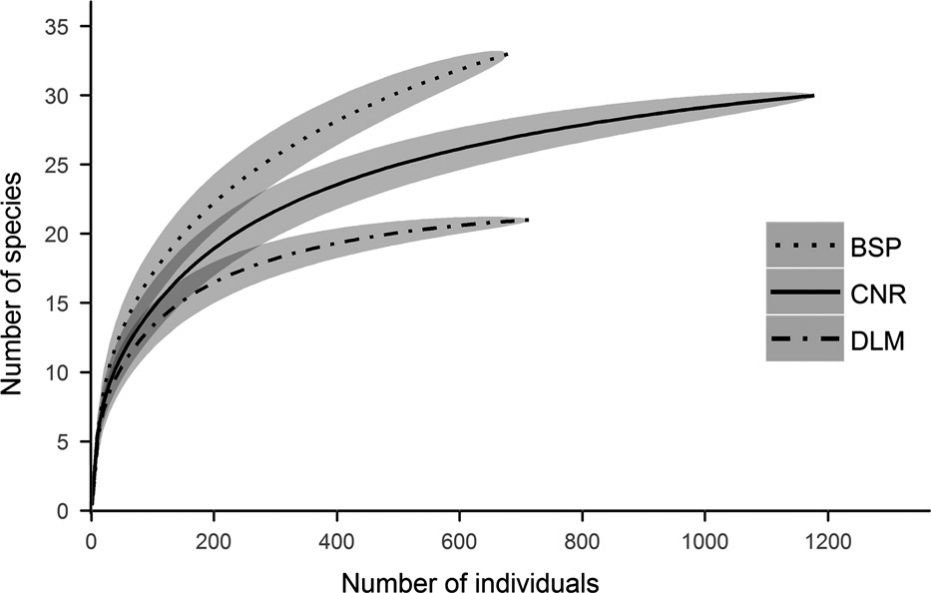
Rarefaction curves of carabids in different regions (shaded areas represent 95% confidence intervals; BSP: Bashang Plateau; CNR: Changbaishan Nature Reserve; DLM: Dongling Mountain).

**Figure 4:**
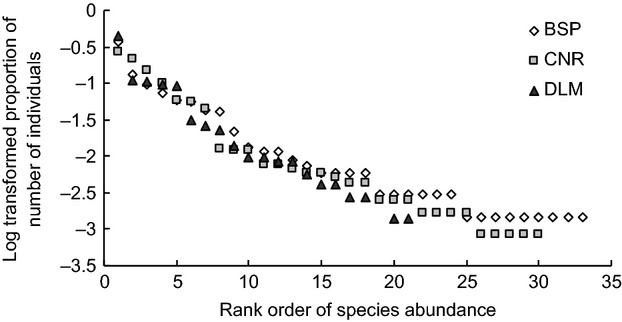
Rank–abundance distribution of carabid species in Changbaishan Nature Reserve (CNR), Dongling Mountain (DLM), and Bashang Plateau (BSP).

A total of 2571 carabids representing 73 species were collected in the plots representing the three study areas. Of these, 1178 individuals representing 30 species were recorded at CNR, 714 individuals representing 20 species at DLM, and 679 individuals representing 33 species, at BSP. Rarefaction curves showed a similar trend to the observed species richness, with BSP having the highest rarefied species richness and DLM reaching the lowest estimated value (Fig. 3). In comparison between the three regions, the dominant ground beetle species take up similar proportions in the assemblages (Fig. 4). In CNR, carabid assemblages were dominated by *Pterostichus vladivostokensis* Lafer, *Pt. orientalis* Motschulsky, and *Pt. interruptus* Dejean, accounting for 62.5% of all individuals. The assemblages in DLM were dominated by *Pt. acutidens* Fairmaire, *Carabus crassesculptus* Kraatz, and *C. manifestus* Kraatz, which accounted for 65.8% of all individuals. At BSP, the three dominant species *Pt. fortipes* Chaudoir, *Pseudotaphoxenus mongolicus* Jedlicka, and *Pt. gebleri* Dejean accounted for 60.1% of the sampled individuals. The pattern of rare species (accounting for less than 1% of the specimens in the samples, and with a log value lower than −2) was different between the three regions: BSP samples contained 21 rare species and CNR had a very similar number of 20 rare species, while samples at DLM only contained 11 rare species (Fig. 4).

### Species composition, turnover, and assemblage similarities

**Figure 5:**
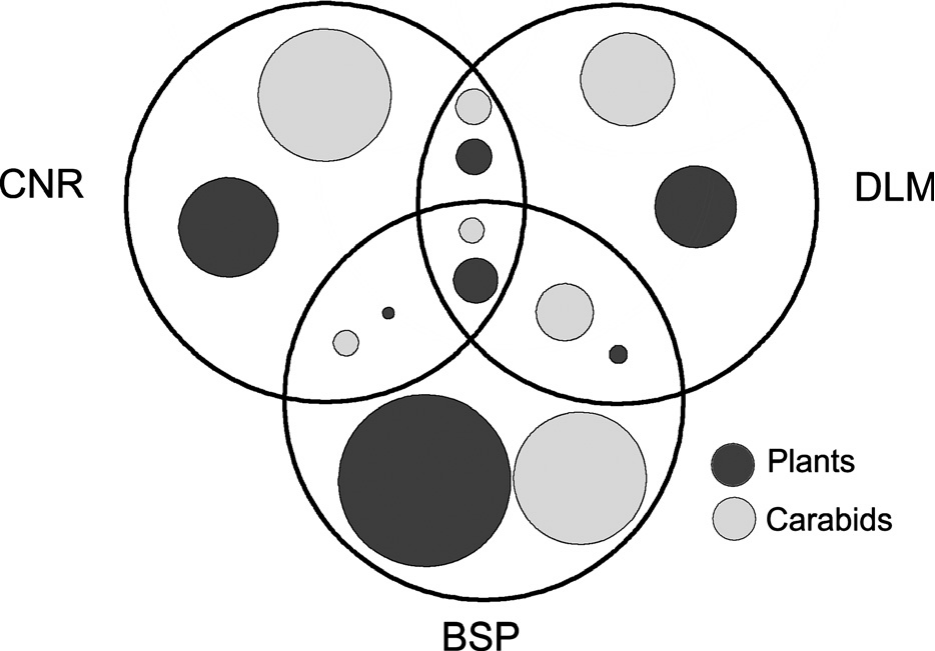
Proportion of shared and unique species between the three study regions, with dot size proportional of the number of species (BSP: Bashang Plateau; CNR: Changbaishan Nature Reserve; DLM: Dongling Mountain).

In relation to species composition, CNR shared more herbaceous plant species with DLM (six species) than with BSP (one species) (Fig. 5). In BSP, 172 of the 184 recorded herbaceous plant species (93%) were unique to these plantation forests, while the proportion of unique species for herbaceous plants in CNR was also high (83%), but much lower in DLM (65%) (Fig. 5). *Impatiens noli-tangere* Linn. was the only herbaceous species that was recorded at all three regions.

For carabids, CNR shared 2 species with DLM and 1 species with BSP. Both CNR and BSP harbored a high number of unique species of carabids (26 species) (Fig. 5), while the overall abundance assemblage structure between these sites was quite different. In total, unique species accounted for 70.6% of the sampled individuals in CNR, while this number decreased to 62% and 36.8% in DLM and BSP, respectively. The five species BSP shared with DLM already accounted for 62% of all specimens caught in BSP. Only one carabid species, *Carabus canaliculatus* Adams, appeared in samples from all three regions, with 52 individuals caught at CNR (4.4%), 10 at DLM (0.14%), and only 4 at BSP (0.6%).

**Figure 6:**
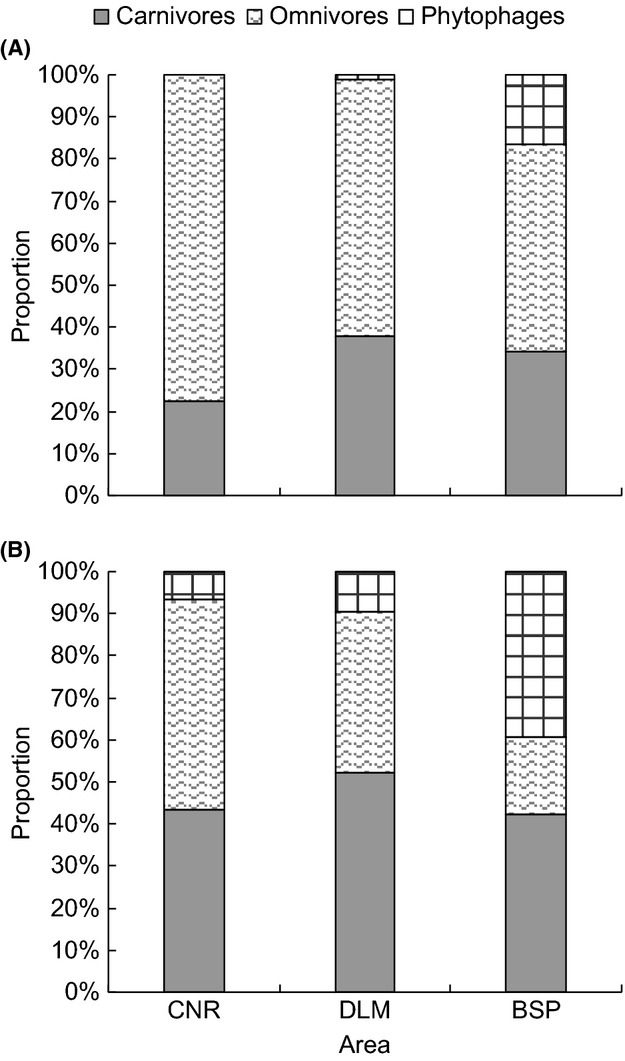
Carabid feeding guild composition in relation to (A) abundance and (B) species richness at different regions (BSP: Bashang Plateau; CNR: Changbaishan Nature Reserve; DLM: Dongling Mountain).

Omnivores were the most abundant carabid feeding guild at all three study regions, with 13 omnivorous species representing 77.3% of all sampled individuals recorded at CNR, 7 species (60.9%) at DLM and 13 species (49.3%) at BSP (Fig. 6). At both DLM (11 species) and BSP (14), however, carnivores were the most species-rich group (Fig. 6). Both CNR and DLM harbored only 2 phytophagous species, each, which also accounting for only 0.2% and 6.7% of the total number of individuals, respectively. In contrast, the 13 phytophagous species sampled at BSP accounted for 39.4% of all individuals in the samples.

**Figure 7:**
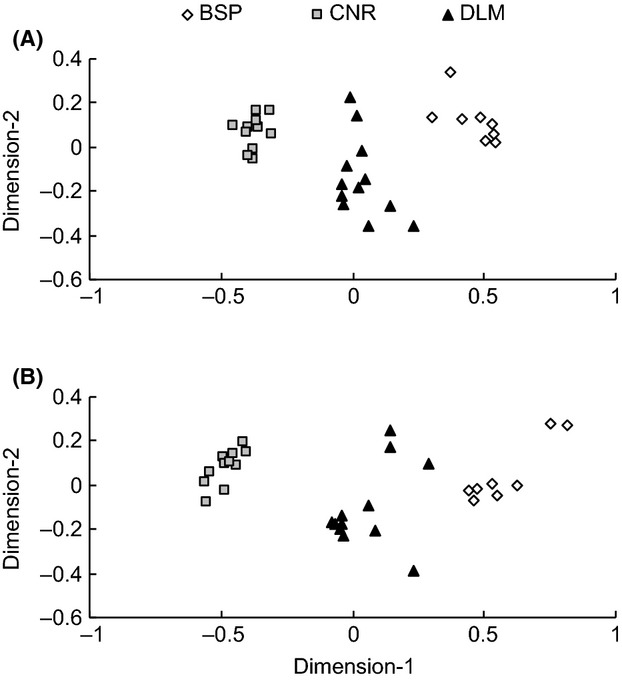
Nonmetric two-dimensional scaling (NMTDS) ordination plots for herbaceous plants based on the “Jaccard” (incident data) dissimilarity matrices (A, stress = 0.09), and based on the CNESS dissimilarity matrices for a minimum sample sizes *m *=* *1 for carabids (B, stress = 0.04) (BSP: Bashang Plateau; CNR: Changbaishan Nature Reserve; DLM: Dongling Mountain).

When comparing the species turnover and assemblage patterns between the three regions, herbaceous plant species composition showed three very distinctive clusters, each representing one of the study regions. Sampling plots at CNR and BSP formed two particularly tight clusters, while plots in DLM were more spread out and positioned between the CNR and BSP clusters (see Fig. 7A). Plant species in DLM had a higher *β*-diversity than in the other two regions, with an average dissimilarity value of 0.84, while the average value was 0.74 and 0.59 for BSP and CNR, respectively.

The species composition pattern for carabids again was clearly differentiated into the three study regions in the ordination diagram. In this diagram, CNR assemblages were still grouped into a denser cluster than BSP and DLM, and DLM again had the highest *β*-diversity, with assemblages again located at an intermediate position between CNR and BSP (Fig. 7B). The average dissimilarity values were 0.77, 0.64, and 0.67 for DLM, CNR, and BSP, respectively. The first axes of the NMTDS ordination plots representing the species turnover in both, herbaceous plants and carabids, were highly correlated with altitude (Pearson correlation, *r* = 0.89 for plants and *r* = 0.92 for carabids, respectively, with *P* < 0.001 in both cases).

## Discussion

### *α*-diversity

Probably the most striking finding presented in this study is the high diversity of herbaceous plant species in the plantation forests of BSP. In this region, the earliest larch plantations were planted in the late 1980s with the aim of forest habitat restoration, and since then, these forest plantations have been well protected. A few previous studies have shown that native timber plantations can increase biodiversity through the provision of opportunities for understory plant regeneration (Ashton et al. 2001; Carnevale and Montagnini 2002). This relates, for example, to increasing topsoil nutrient contents, facilitating the influx of site-sensitive tree, shrub, and herb species (Grubb 1995). Another possible reason is that trees in plantation forests at BSP are less densely planted than trees in naturally generated secondary forest (DLM) and mature forest (CNR) ecosystems. This would result in less canopy closure in the BSP forests, allowing for a higher species richness and density in understory plants (Lemenih et al. 2004) in the larch plantations. This factor might also partly explain the high species richness for carabids at BSP, as some carabid species prefer areas with open canopies (Butterfield et al. 1995; Humphrey et al. 1999; Taboada et al. 2008; Lange et al. 2014). This is also in line with findings from Europe that younger forests with less canopy closure harbor higher carabids species richness (Taboada et al. 2008).

The high diversity of herbaceous plants could additionally relate to the fact that forest plantations in BSP were established on former grassland habitats. These habitats were relatively weakly disturbed prior to plantation establishments and harbored typical, highly diverse local steppe plant assemblages, resulting in the plantations now harboring a mixture of grassland and undergrowth forest species. This is also reflected in the great similarity in carabid species composition between forests and grasslands in this region (Liu et al. 2012). The observed high diversity of carabids could accordingly be contributed to the high diversity of herbaceous plants in BSP, which is likely to result in a wide range of microclimatic conditions and humus layer depths as well as a very heterogeneous vegetation structure, hence providing suitable habitats for a wide range of ground-dwelling invertebrates (Carnus et al. 2006).

### Species composition and turnover patterns

In spite of the substantial geographical distance, elevational and local climatic differences between the three study regions, there is still some overlap in herbaceous plant and insect species. Interestingly, most species of carabids shared between CNR and DLM were nondominant species. The proportion of unique species in BSP (78.8%) did not differ much from that at CNR (86.7%), but the abundance of the unique species in BSP was very low in comparison with the other two regions. This results from the dominant carabid species at BSP also occurring at DLM. Plantation forests have previously been identified as potential habitats of endangered species, such as *Holcaspis brevicula* Butcher recorded in New Zealand pine plantation forests (Brockerhoff et al. 2005), and three nationally spare species in the genera *Trechus* and *Pterostichus* in pine and spruce plantation forests in Britain (Jukes et al. 2001). It can therefore be speculated that plantation forests in BSP might also potentially sustain suitable habitats for rare species of carabids, although the knowledge base for ground beetles in China is insufficient for a detailed evaluation of the occurrence patterns of nationally or regionally rare species or to assign a threat status to the vast majority of Chinese carabids. Nevertheless, for the plantations on degraded steppe environments at BSP, phytophagous beetles are likely to have remained in the open forests. These species might be in particular danger because of the negative effect of increasing canopy closure (Butterfield et al. 1995; Jukes et al. 2001; Taboada et al. 2008) with the increase in forest age. At the same time, the mature forests at CNR harbor the highest number of carabid forest specialist species, indicating that these forests might provide unique food resources and microclimatic conditions for carabid specialists, which illustrates the special conservation significance of these mature forests.

In relation to turnover patterns, the high turnover rates observed between some of the secondary forest plots on Dongling Mountain for both beetles and plants are likely to reflect the different habitat conditions encountered in the three distinct forest types investigated in this region. This variation between individual plots contrasts with the much finer microhabitat mosaics encountered in the mature mixed forests on Changbai Mountain and with the more homogeneous habitat conditions encountered under planted larch forests. It is nonetheless noteworthy that particularly the ground beetle assemblages appear to be differentiated so distinctly, despite all plots being situated within relatively close vicinity to each other and connected by uninterrupted forest habitats. This underlines the sensitivity of both the local ground beetle species pool and of the understory plants to the subtle differences in environmental conditions between the investigated broad-leaved forest habitats. This also underlines the importance of tree species choice (here of larch) in the large-scale afforestation programs across China, which will have severe implications for the assemblage structure and biodiversity encountered in the resulting plantation forest habitats.

The difference in species assemblages of carabid beetles also reflects differences in function and services of the three forest regions. Due to the high diversity of herbaceous plants at BSP, it is unsurprising that phytophagous carabids account for a higher proportion of species in comparison with the other two regions. Previous studies have reported positive relationship between the abundance of phytophagous carabids and plant species richness (Harvey et al. 2008), as high plant diversity can potentially provide more food resources for those herbivore species (Haddad et al. 2001). The high proportion of phytophagous carabids at BSP is furthermore in line with this forest being the least mature, and the high overall species richness cannot be taken as indicative of ecosystem stability in this plantation region. In relation to the classification of phytophagous species, it should be pointed out that 10 of the 13 species at BSP classed as phytophagous were of the genus *Harpalus*. Members of this genus were classified as phytophages according to Harvey et al. (2008), while other studies have also classified them as omnivores (ElSayed and Nakamura 2010).

The high proportion of predators in contrast to phytophages is seen as indicative of a more complex food web structure commonly linked to mature, stable ecosystems. The mature forest of CNR contains substantial, varying amounts of litter and woody debris that might harbor abundant prey species (Negro et al. 2014), hence supporting a higher proportion of predatory carabids. In CNR, carabid assemblages were composed chiefly of carnivores and omnivores, reflecting the stable, mature ecosystems in this region. In terms of their overall ecosystem function, carabids at DLM and particularly at CNR therefore occupy potentially higher trophic levels than the species encountered at BSP. At the latter site, the carabid assemblages can directly impact on the vegetation composition due to herbivore pressures on plant species, whereas in the other regions, the assemblages will more efficiently fulfill pest control functions relating to insects and other invertebrate species.

## Conclusion

Our study regions all harbored relatively high levels of *α*-diversity for carabid beetles in comparison with temperate forests in Europe (e.g., Avgın 2006; Skalski et al. 2011), suggesting a high conservation value of the forests in the wider region. It should also be noted in this regard that criticism raised about China's plantations causing an overall decrease of species diversity primarily in the vegetation (e.g., Cao 2008; Cao et al. 2010a) was chiefly focused on fast-growing, water-inefficiency *Populus* spp. plantations. For conservative purposes, different choices of tree species used in plantations commonly result in very different ecological outcomes for forest ecosystem communities. Our results indicate that the wide range of protected larch plantation forests in northern China might potentially be of considerable conservation value, not only for vegetation restoration and in preventing soil erosion and land degradation, but also in sustaining high biodiversity levels for ground-dwelling arthropods such as carabids. Nonetheless, it should be noted that high diversity in itself is insufficient in indicating a “good” forest ecosystem, as species composition also requires consideration. The few remaining large mature forests in temperate China such as that studied at the CNR can potentially provide crucial source areas for a high proportion of predatory carabid species, indicating a well-developed, complex ecosystem. Therefore, China's remnant mature forests require specific conservation attention.

## Supporting Information

**Appendix S1:** Species list of ground beetles at Changbaishan Nature Reserve (CNR), Dongling Mountain (DLM), Bashang Plateau (BSP).
