# Supplementary material for: Diversity patterns of ground beetles and understory vegetation in mature, secondary, and plantation forest regions of temperate northern China
Source: Ecol Evol. 2015 Jan 7;5(3):531–42. doi: 10.1002/ece3.1367 (PMC4328759; doi:10.1002/ece3.1367)
Supplement: Supplementary file 1 [file ece30005-0531-sd1.doc]

**Appendix** Species list of ground beetles at Changbaishan Natural Reserve (CNR), Dongling Mountain (DLM), Bashang Plateau (BSP).

| **Species names** | **CNR** | **DLM** | **BSP** | **Total** |
| --- | --- | --- | --- | --- |
| *Agonum* sp1 | 0 | 0 | 28 | 28 |
| *Agonum* sp2 | 9 | 0 | 0 | 9 |
| *Agonum* sp3 | 0 | 0 | 8 | 8 |
| *Amara communis* Panzer 1797 | 0 | 0 | 2 | 2 |
| *Amara macronota* Solsky 1875 | 0 | 0 | 4 | 4 |
| *Amara* sp | 0 | 0 | 2 | 2 |
| *Asaphidion semilucidum* Motschulsky 1862 | 0 | 2 | 0 | 2 |
| *Asaphidion* sp | 1 | 0 | 0 | 1 |
| *Bembidion difforme* Motschulsky 1844 | 0 | 0 | 1 | 1 |
| *Carabus billbergi* Mannerheim 1827 | 14 | 0 | 0 | 14 |
| *Carabus brandti* Faldermann 1835 | 0 | 0 | 2 | 2 |
| *Carabus canaliculatus* Adams 1812 | 52 | 10 | 4 | 66 |
| *Carabus crassesculptus* Kraatz 1881 | 0 | 80 | 15 | 95 |
| *Carabus fraterculus* Reitter 1895 | 3 | 0 | 0 | 3 |
| *Carabus granulatus* Linne 1758 | 8 | 0 | 0 | 8 |
| *Carabus kruberi* Fischer 1822 | 0 | 1 | 38 | 39 |
| *Carabus latreillei* Fischer 1822 | 0 | 0 | 2 | 2 |
| *Carabus manifestus* Kraatz 1881 | 0 | 74 | 0 | 74 |
| *Carabus seishinensis* Lapouge 1931 | 70 | 0 | 0 | 70 |
| *Carabus sui* Imura & Zhou 1998 | 0 | 2 | 0 | 2 |
| *Carabus vietinghoffi* Adams 1812 | 15 | 0 | 0 | 15 |
| *Carabus vladimirskyi* Dejean 1830 | 0 | 66 | 29 | 95 |
| *Chlaenius pallipes* Gebler 1823 | 0 | 0 | 1 | 1 |
| *Cychrus morawitzi* Gehin 1863 | 14 | 0 | 0 | 14 |
| *Dolichus halensis* Schaller 1783 | 0 | 0 | 4 | 4 |
| *Harpalus brevicornis* Germar 1824 | 0 | 0 | 5 | 5 |
| *Harpalus bungii* Chaudoir 1844 | 0 | 0 | 49 | 49 |
| *Harpalus chalcentus* Bates 1873 | 0 | 0 | 1 | 1 |
| *Harpalus coreanus* Tschitscherine 1895 | 0 | 3 | 0 | 3 |
| *Harpalus eous* Tschiterscherine 1901 | 0 | 0 | 4 | 4 |
| *Harpalus laevipes* Zetterstedt 1828 | 0 | 6 | 0 | 6 |
| *Harpalus lumbaris* Mannerheim 1825 | 0 | 0 | 1 | 1 |
| *Harpalus melaneus* Bates 1878 | 0 | 0 | 39 | 39 |
| *Harpalus roninus* Bates 1873 | 0 | 0 | 2 | 2 |
| *Harpalus simplicidens* Schauberger 1929 | 0 | 0 | 1 | 1 |
| *Harpalus sinicus* Hope 1845 | 0 | 0 | 1 | 1 |
| *Harpalus suensoni* Kataev 1997 | 0 | 0 | 1 | 1 |
| *Harpalus ussuricus* Mlynar 1979 | 1 | 0 | 0 | 1 |
| *Harpalus xanthopus* Gemminger & Harold 1868 | 1 | 0 | 0 | 1 |
| *Leistus niger* Gebler 1847 | 67 | 0 | 0 | 67 |
| *Martyr alter* Semenov & Znojko 1929 | 0 | 0 | 2 | 2 |
| *Notiophilus impressifrons* Morawitz 1862 | 0 | 3 | 0 | 3 |
| *Panagaeus davidi* Fairmaire 1887 | 0 | 1 | 0 | 1 |
| Pristosia sp1 | 5 | 0 | 0 | 5 |
| Pristosia sp2 | 5 | 0 | 0 | 5 |
| Pristosia sp3 | 0 | 6 | 0 | 6 |
| Pristosia sp4 | 0 | 0 | 1 | 1 |
| *Pseudotaphoxenus mongolicus* Jedlicka 1953 | 0 | 22 | 89 | 111 |
| *Pseudotaphoxenus rugipennis* Fald 1836 | 0 | 0 | 9 | 9 |
| *Pterostichus acutidens* Fairmaire 1889 | 0 | 316 | 0 | 316 |
| *Pterostichus adstrictus* Eschscholtz 1823 | 117 | 69 | 0 | 186 |
| *Pterostichus comorus* Jedlicka 1932 | 2 | 0 | 0 | 2 |
| *Pterostichus fortipes* Chaudoir 1850 | 0 | 16 | 253 | 269 |
| *Pterostichus gebleri* Dejean 1831 | 0 | 0 | 66 | 66 |
| *Pterostichus gibbicollis* Motschulsky 1844 | 3 | 0 | 0 | 3 |
| *Pterostichus horvatovichi* Kirschenhofer 1991 | 6 | 0 | 0 | 6 |
| *Pterostichus interruptus* Dejean 1828 | 176 | 7 | 0 | 183 |
| *Pterostichus jankowskyi* Tschitscherine 1897 | 7 | 0 | 0 | 7 |
| *Pterostichus mandzhuricus* Lustshnik 1916 | 2 | 0 | 0 | 2 |
| *Pterostichus microcephalus* Motschulsky 1860 | 1 | 0 | 1 | 2 |
| *Pterostichus nigrita* Paykull 1790 | 2 | 0 | 0 | 2 |
| *Pterostichus orientalis* Motschulsky 1844 | 256 | 0 | 0 | 256 |
| *Pterostichus* sp | 3 | 0 | 0 | 3 |
| *Pterostichus subovatus* Motschulsky 1860 | 0 | 7 | 0 | 7 |
| *Pterostichus vladivostokensis* Lafer 1979 | 319 | 0 | 0 | 319 |
| *Synuchus agonus* Tschitscherine 1895 | 7 | 0 | 0 | 7 |
| *Synuchus nordmanni* Morawitz 1862 | 0 | 0 | 8 | 8 |
| *Synuchus* sp1 | 0 | 19 | 0 | 19 |
| *Synuchus* sp2 | 0 | 0 | 6 | 6 |
| *Synuchus* sp3 | 9 | 0 | 0 | 9 |
| *Synuchus* sp4 | 1 | 0 | 0 | 1 |
| *Trichotichnus coruscus* Tschitscherine 1895 | 2 | 0 | 0 | 2 |
| *Trichotichnus* sp | 0 | 4 | 0 | 4 |
| **Total number of species** | **30** | **20** | **33** | **73** |
| **Total number of individuals** | **1178** | **714** | **679** | **2571** |
